# Thyroid Dysfunction and Autoantibodies Association with Hypertensive Disorders during Pregnancy

**DOI:** 10.1155/2012/742695

**Published:** 2012-07-16

**Authors:** Azin Alavi, Khadijeh Adabi, Sepideh Nekuie, Elham Kazemi Jahromi, Mehrdad Solati, Alireza Sobhani, Hoda Karmostaji, Alireza Shahab Jahanlou

**Affiliations:** ^1^Obstetrics and Gynecology Department, Shariati Hospital, Infertility and Reproductive Health Research Center, Hormozgan University of Medical Sciences, Naser Boulevard, Bandarabbas 7917746345, Iran; ^2^Obstetrics and Gynecology Department, Women Hospital, Tehran University of Medical Sciences, North Ostad Nejatolahi Avenue, Karim Khan Street, Tehran, Iran; ^3^Pathology Department, Shariati Hospital, Hormozgan University of Medical Sciences, Naser Boulevard, Bandarabbas, Iran; ^4^Endocrinology Department, Shariati Hospital, Hormozgan University of Medical Sciences, Naser Boulevard, Bandarabbas 7917746345, Iran; ^5^Obstetrics and Gynecology Department, Shariati Hospital, Hormozgan University of Medical Sciences, Naser Boulevard, Bandarabbas, Iran; ^6^Health Education Unit, Hormozgan University of Medical Sciences, Naser Boulevard, Bandarabbas, Iran

## Abstract

*Background*. Thyroid dysfunction and autoimmunity are relatively common in reproductive age and have been associated with adverse health outcomes for both mother and child, including hypertensive disorders during pregnancy. *Objective*. To survey the relation between thyroid dysfunction and autoimmunity and incidence and severity of pregnancy-induced hypertensive disorders. *Method*. In this case control study 48 hypertensive patients in 4 subgroups (gestational hypertension, mild preeclampsia, severe preeclampsia, eclampsia) and 50 normotensive ones were studied. The samples were nulliparous and matched based on age and gestational age and none of them had previous history of hypertensive or thyroid disorders and other underlying systemic diseases or took medication that might affect thyroid function. Their venous blood samples were collected using electrochemiluminescence and ELISA method and thyroid hormones and TSH and autoantibodies were measured. *Results*. Hypertensive patients had significant lower T3 concentration compared with normotensive ones with mean T3 values 152.5 ± 48.93 ng/dL, 175.36 ± 58.07 ng/dL respectively. Anti-TPO concentration is higher in control group 6.07 ± 9.02 IU/mL compared with 2.27 ± 2.94 IU/mL in cases. *Conclusion*. The severity of preeclampsia and eclampsia was not associated with thyroid function tests. The only significant value was low T3 level among pregnancy, induced hypertensive patients.

## 1. Introduction

Thyroid dysfunction and autoimmunity are relatively common among women of reproductive age with prevalence of 2-3% during pregnancy. Although during reproductive age thyroid autoantibodies are found in 5–15% of women, they are not necessarily accompanied by thyroid dysfunction [1]. 

Thyroid disorders during pregnancy are associated with adverse health outcomes for both mother and child, including increased risk of miscarriage, gestational hypertension, preterm delivery, placental abruption, low birth weight and fetal death [2, 3]. 

Association of mild maternal thyroid insufficiency has been reported with delayed neuropsychological development in neonate and child [4–6]. It has been suggested that antithyroid antibodies are independent markers of at risk pregnancy [7]. Presence of antithyroid antibodies can result in increased risk of miscarriage, gestational diabetes mellitus, postpartum thyroiditis, permanent hypothyroidism, depression, and impaired child development [8–10]. 

It remains to be established whether screening and subsequent treatment will improve clinical outcome, and which risk factors contribute to the complications resulting from thyroid abnormalities. We undertook this study in order to determine the association of thyroid diseases and autoantibodies with gestational hypertension, eclampsia, and preeclampsia in Iranian pregnant women.

## 2. Materials and Methods 

This case control study included 18- to 35-year-old nulliparous pregnant women with single pregnancy, and gestational age of 20 weeks or more (based on first trimester sonography) who presented at Shariati hospital, Hormozgan university of medical sciences, Bandarabbas, Iran, in 2010. This study was approved by ethics committee of medical faculty. All the patients signed an informed consent. The study group consisted of 50 cases, and 50 controls that were selected randomly from a population of patients admitted with a diagnosis of gestational hypertension, the controls were recruited from normotensive pregnant women admitted for pregnancy termination. Needed information was collected through careful history and complete physical examination. Blood pressure of all cases were checked and complete prenatal laboratory studies including urine analysis, blood glucose, BUN, and ceratinin were done before 20th week of gestation, in order to exclude those with chronic hypertension and other systemic disorders and to diagnose preeclampsia and gestational hypertension more accurately.

Blood pressure was measured by mercury sphygmomanometers in a sitting position with the arm supported at the level of the heart. The cuff was inflated to a pressure approximately 30 mmHg greater than systolic, as estimated from the disappearance of the pulse in the brachial artery by palpation to avoid potential problems with an auscultatory gap. All pregnant women with a history of thyroid disease (hypo, or hyperthyroidism, goiter, grave's, autoimmune, and toxic nodular thyroiditis, etc.), systemic disorders (diabetes, renal diseases, epilepsy or other convulsive disorders, chronic hypertension, autoimmune and collagen vascular diseases) multiple pregnancy, and history of medication affecting thyroid function (Levothyroxine, Metimazole, PTU, and other anti thyroid medications) were excluded.

The diagnosis of hypertension was confirmed in hospital as blood pressure of more than 140/90 mmHg recorded on two occasions at least 6 hours apart. Women with hypertension after 20th week of gestation which became normal within the first 24-hours after labor and had no proteinuria on dipstick test or 24-hour urine collection were considered to have gestational hypertension and those with hypertension after 20th week of gestation and 300 mg or more (less than 2 grams) proteinuria in 24-hour urine sample or equal or more than 1+ proteinuria on 2 separate dipstick clean catch midstream urine samples with a minimum 6-hour interval were considered to have mild preeclampsia and those with systolic blood pressure equal or more than 160 mmHg or diastolic blood pressure equal or more than 110 on two occasions, or proteinuria equal or more than 2 grams in 24-hour urine sample or any of severe preeclampsia's criteria including oliguria (urine less than 500 cc per 24 h), thrombocytopenia (platelet count less than 100,000), raised liver enzymes aspartate aminotransferase (SGOT) > 50 U/L or alanine aminotransferase (SGPT) > 60 U/L, epigastric or RUQ pain, pulmonary edema, visual or brain function disturbance (severe headache, diplopia, blurred vision) or intrauterine growth restriction of the fetus were considered as severe preeclampsia and those with seizures as eclampsia in our study.

### 2.1. Laboratory Analysis

 Before labor, 5 CC blood sample was obtained from an antecubital vein of each case. T3, T3RU, TSH, TT4 were assayed with ELISA method by DIAPLAUSE kit and antiTPO, free T4 and anti TG levels were assayed by luminance method by DIASORIAN kit. All biochemical measurements were performed by RIA. After getting the results, each patient's type of thyroid dysfunction was determined by means of TSH and FT4 levels as following: TSH > 4.5 mu/mL and normal FT4I was included as subclinical hypothyroidism, TSH > 4.5 and low FT4I as clinical hypothyroidism, TSH < 0.4 and high FT4I as hyperthyroidism and otherwise as euthyroid in our study [11]. At the same time, high levels of anti TG or anti TPO or both were considered as positive autoantibodies and otherwise, negative autoantibodies. 

Normal ranges were defined due to the laboratory's normal ranges according to the kits they used and are as the following: Anti-TPO: 1–16 IU/mL, Anti TG: 5–100 IU/mL TSH: 0.4–4.5 micro-IU/mL, T3: 77.8–355 ng/dL, FT4I: 1.3–4.6 (RATIO), T3RU: 25–35 U%, Total T4:4.8–12.6 micro gram/dL, free T4: 0.8–1.7 micro g/dL.

### 2.2. Statistical Analysis

 The collected data were analyzed using the Statistical Package for the Social Sciences (SPSS) version 15.0. Descriptive data were presented as mean and standard deviation (SD), and or percentage. Differences between quantitative data were evaluated by using ANOVA and Bonferroni post hoc test. Significant level of *P* value is considered less than 0.05. 

## 3. Results

 This case control study was performed on 98 pregnant women (48 women with gestational hypertension, and 50 healthy women), 2 cases were excluded, one due to receiving massive transfusion before sampling and the other due to lysis of the blood sample.

Demographic data of cases and controls are presented in Table 1. Average gestational age of control group was (38.26 ± 1.87 weeks) and case group was (37.02 ± 3.3 weeks) which gestational age was homogenous in both group (*P* = 0.121), also there was no significant statistical difference among the 4 groups of cases (Table 1). Mean T3 level and mean anti TPO level were significantly lower in case group with *P* = 0.038 *P* = 0.007, respectively. There were no other significant differences regarding other measured hormone levels including: Total T4, free T4, T3RU, FTI, TSH, Anti TG (Table 2). No significant difference in measured values was observed among the 4 groups of cases including gestational hypertension, mild preeclampsia, severe preeclampsia and eclampsia (Table 3). Five women among cases and 5 among controls had subclinical hypothyroidism. Of those 5 cases 2 had mild preeclampsia and 3 had severe preeclampsia that represents no statistically significant difference. Autoantibody was positive in 7 women, 5 of which were from control group and 2 from case group. This was not statistically significant.

From these 98 pregnancies 94 resulted in live births and 4 resulted in intrauterine fetal deaths (IUFD), 2 IUFD were related to pregnancies associated with gestational hypertension and the other 2 with severe preeclampsia. The mean 1st and 5th APGAR scores of neonates in case groups were 7.88 ± 1.61 and 9.15 ± 1.41 respectively, and in control group 8.54 ± 0.81 and 9.76 ± 0.55. There was no statistically difference between them. Mean neonatal weight regarding gestational age was 2661.14 ± 795.18 grams in case group and 2913.4 ± 448.72 grams in control group with no statistically significant difference, there was also no significant difference between neonates of the 4 case groups. Mean Apgar score of 1st minute in neonates of case group was 7.88 ± 1.61 and in neonates of control group was 8.54±0.81 with statistically significant difference (*P* = 0.013). Mean Apgar score of 5th minute in neonates of case group was 9.15 ± 1.41 and in neonates of control group was 9.76 ± 0.55 with statistically significant difference (*P* = 0.007). Mean Apgar score of 1st and 5th minute in the 4 case groups had no statistically significant difference. 

## 4. Discussion

The relationship between thyroid function and obstetrical complications is a matter of concern nowadays. Although several studies have been performed to determine the association between hypothyroidism (overt or subclinical), or presence of thyroid autoantibodies and maternal and fetal adverse consequences, there is still controversy in this regard. Preeclampsia and eclampsia are severe complication of pregnancy and have an important role in pregnancy outcome [12]. As it is known, in normal pregnancies the increased level of estrogen causes higher levels of TBG and these results in low levels of T3RU. In some studies it is shown that dysfunction of the placenta may cause low levels of estrogen that may cause low levels of T3, T4 and TBG [13], also in toxic patients decreased TBG causes low level of T4 and this causes decreased conversion of T4 to T3 in the liver [14], on the other hand any inflammatory or other acute process such as pregnancy can cause impaired thyroid function tests without a history of thyroid disorder. This is called sick euthyroid syndrome and the most frequent type that we see is low-T3 syndrome (low levels of T3 in presence of normal TSH and T4 levels) [13]. 

Other than an association between low levels of T3 and preeclampsia and eclampsia, we found no other associations between thyroid hormones or thyroid autoantibodies and incidence or severity of preeclampsia and eclampsia. This isolated low-T3 level (mild biochemical hypothyroidism) may be due to placental dysfunction or normal reaction of the body to pregnancy as mentioned above. In 2010 Sahu et al. studied 633pregnant women and found a positive relationship between overt and subclinical hypothyroidism and significant adverse effects on maternal and fetal outcomes; including pregnancy-induced hypertension, intrauterine growth restriction, and intrauterine demise in India [15]. This was not congruent with the results reported by Wolfberg et al., which showed that hypothyroid patients may be at increased risk for preeclampsia even after treatment [16]. 

In Tehran, Iran, 2004, Larijani et al. have done a study to evaluate thyroid hormone alteration in preeclamptic pregnant women on 39 preeclamptic patients and 42 healthy controls and reported increased TSH levels and decreased free and total levels of T4 and T3 compared to healthy controls [14], that supports Kaya et al.'s [17] and Tolino et al.'s [18] studies. This was just one year after Ramezani Tehrani and colleagues performed a study on 40 preeclamptic patients and 40 healthy women and suggested that mild biochemical hypothyroidism is found in proteinuric preeclampsia and the concentration of T3 seem to reflect the severity of preeclampsia [19]. None of them studied thyroid autoantibodies. The Anti-TPO and Anti-TG-level evaluation and their relationship determination with preeclampsia and eclampsia is what make, our study different from other studies that were previously performed in Iran. In 2008 Moncef Feki et al. performed a study on 1519 pregnant women in Tunis and found that women with positive anti TPO have a trend toward higher prevalence of gestational hypertension and past pregnancy loss particularly late abortion and fetal death [20]. Negro et al. showed in a study on 984 pregnant women in 2006, that euthyroid pregnant women who are positive for TPO Ab develop thyroid function impairment which is associated with increased risk of miscarriage and premature deliveries [21]. These two studies agree with Federico Mecacci's study result in 2000; that showed a positive relation between Anti-TPO and Anti TG and preeclampsia [22]. In contrast Männistö et al. studied 9247 pregnant women in 2010 and found no difference in incidence rate of gestational hypertension between thyroid dysfunction group and reference group or between TPO antibody positive and negative mothers [23]. 

Likewise in our study incidence of gestational hypertension, mild or severe preeclampsia and eclampsia between mothers with hypothyroidism and control group or autoantibody positive and negative mothers had no significant difference (other than the low-T3 level's association with gestational hypertension, mild or severe preeclampsia and eclampsia). Although this finding does not support some studies it does agree with the current position of American societies and authors against systematic screening for thyroid diseases during pregnancy [24]. As our study's sample size is small, further clinical studies must be conducted to reach to a unique agreement on the clinical consequences and outcomes of pregnancies with thyroid disorders.

## 5. Conclusions

 The severity of preeclampsia and eclampsia was not associated with thyroid function tests. The only significant value was low-T3 level among pregnancy, induced hypertensive patients. According to this study, we do not recommend screening of hypertensive pregnant women for thyroid dysfunction, regardless of their history. Due to small sample size of our study, further clinical studies must be conducted to reach to a unique agreement.

## Figures and Tables

**Table 1:** Demographic features of healthy pregnant women and women with hypertensive disorders during pregnancy.

	Control	Gestational HTN	Mild pre-eclamsia	Severe pre-eclampsia	Eclampsia	*P* value
Number	50	5 (10.4%)	16 (33.3%)	24 (50%)	3 (6.2%)	
Age (year)	22.18 ± 3.14	21.6 ± 4.27	22.12 ± 2.5	22.38 ± 4.17	23 ± 3.6	NS
Gestational age (weeks)	38.26 ± 1.87	37 ± 4.12	37.75 ± 3.02	36.71 ± 3.35	35.67 ± 4.04	*P* = 0.121

NS: not significant (*P* value > 0.05).

**Table 2:** Average levels of thyroid hormones and thyroid auto antibodies in cases and controls.

Thyroid hormone	Case (*n* = 48)	Control (*n* = 50)	*P* value
Anti TPO	2.27 ± 2.94	6.07 ± 9.02	*P* = 0.007
Anti TG	8.14 ± 8.13	8.75 ± 1.2	NS
TSH	2.03 ± 1.7	2.17 ± 1.44	NS
T3	152.5 ± 48.93	175.36 ± 58.07	*P* = 0.038
FT4I	2.04 ± 0.57	2.15 ± 0.48	NS
T3RU	23.38 ± 1.98	22.87 ± 2.71	NS
TOTAL T4	8.72 ± 2.2	9.39 ± 1.71	NS
FREE T4	1.28 ± 2.11	1 ± 1.3	NS

NS: not significant (*P* value > 0.05).

**Table 3:** Thyroid hormone levels in women with gestational hypertension, mild-severe preeclampsia, and eclampsia.

Thyroid hormone	Gestational HTN	Mild preeclampsia	Severe preeclampsia	Eclampsia	*P* value
ANTI TPO	2.47 ± 1.35	3.08 ± 4.83	1.83 ± 0.93	1 ± 0	0.511
ANTI TG	6.05 ± 1.07	9.33 ± 1.11	8.13 ± 7.18	5.34 ± 0.3	0.550
TSH	1.51 ± 47.59	1.99 ± 1.57	2.18 ± 1.96	1.86 ± 1.96	0.956
T3	152 ± 47.59	144.98 ± 41.71	159.66 ± 55.77	136.17 ± 38.21	0.141
FT4I	1.95 ± 0.11	2.01 ± 0.32	2.09 ± 0.76	1.9 ± 0.45	0.090
T3RU	22.49 ± 1.82	23.18 ± 2.37	23.69 ± 1.84	23.45 ± 1.22	0.631
TT4	8.7 ± 0.58	8.7 ± 1.32	8.8 ± 2.9	8.06 ± 1.57	0.992
FT4	0.81 ± 0.11	0.93 ± 0.18	1.25 ± 2.23	4.17 ± 5.72	0.301
